# Does noise pollution influence modal choices? A random forest application

**DOI:** 10.1371/journal.pone.0325249

**Published:** 2025-06-23

**Authors:** Alessia Calafiore, Ki Tong

**Affiliations:** Edinburgh School of Architecture and Landscape Architecture, University of Edinburgh, Edinburgh, United Kingdom; AUM: American University of the Middle East, KUWAIT

## Abstract

This work investigates the relationship between noise pollution and modal choices exploring and comparing two different urban contexts: Greater London and Brisbane. To achieve this, data on commuting flows by mode of transport and estimated noise pollution have been obtained and combined with measures to characterise the built environment which demonstrated to have an influence on modal choices. Random forest models have shown very good performances in solving classification problems to predict transport modes and allow the exploration of non-linear relationships between the predicted classes and explanatory variables. Two random forest models have been tuned, trained and tested to investigate the association between modal choices and contextual variables, including noise pollution, in Greater London and Brisbane. Results have shown that noise levels play a role in predicting modal choices in Greater London, while the characteristics of the built environment are more relevant when predicting modal choices in Brisbane. Furthermore, we find that walking and cycling, despite being both active travel modes, are influenced by very different factors, with cycling displaying patterns more similar to those characterising driving. Evidence showing the varying relationships between walking and cycling with contextual variables, e.g. noise levels, building and street density, presence of amenities can inform more targeted policies to encourage active travel.

## Introduction

Encouraging modal shifts towards active travel is central to making cities healthier and more sustainable (e.g. [1]). Increased walking and cycling are demonstrated to bring about several benefits to both our physical and mental health [2–5] and play a key role in reducing air pollution in cities [6].

In the last decades, data-driven approaches have been developed to measure the level of walkability and bikeability in cities [7–9] with the ambition to inform urban planning [10–12]. Evidence shows that higher accessibility, a greater number of destinations, and a higher land use mix [13–17] can determine people’s willingness to walk or cycle. This work builds upon these studies and investigates, along with known determinants of active travel, the role of noise pollution [18,19].

Until now, noise pollution has gained little attention in the context of characterising how walkable or bikeable places are [19,20], despite people walking and cycling are significantly exposed to noise pollution [21–23]. Leveraging travel behaviour data on commuting obtained from the UK and Australian Censuses, this research aims to provide new insights into the role noise pollution plays in influencing modal choices.

The association between noise, urban forms and functions and travel behaviours is explored with a random forest model, a machine learning approach that has shown to be performing extremely well in the context of identifying the likelihood of modal choices [24,25]. Notably, the appeal in using a random forest model compared to multinomial logistic regression for multiclass classification comes from its ability to capture more complex and non-linear relationships between variables, which are very common while describing human behaviours. Models have been trained, tuned and tested to predict the mode of transport choices learning from commuting patterns, where each trip is classified by transport mode and summarises the level of noise and the main characteristics of the built environment commuters have been exposed to during the trip. The relationships between the probabilities of classifying a trip as being travelled by walk, bicycle or car and the level of noise exposure can be explored based on two measures: the variable importance, which shows how relevant each variable is in order for the model to make the final prediction; and partial dependence plots, which enable a more in-depth interpretation of how the model associates probabilities to a mode of transport in relation to contextual variables, such as noise levels, urban forms and function [26,27].

Comparing to existing works this study develops novel theoretical and methodological angles, providing a new set of evidence to inform context-dependent urban active transport policies. From a theoretical perspective most of the existing studies have either focused solely on walking [28] or cycling [29], or have considered them the same mode under the definition of active travel [30–32]. In this study walking and cycling are considered separately with the focus of uncovering the specificities of each of them. From a methodological perspective, this work develops an approach where the factors influencing travel behaviours are considered throughout the trips’ itineraries commuters undertake, which are computed with a multimodal router, rather than analysing the characteristics of either origins or destinations [24]. Analysing the interactions between noise pollution, urban forms, and urban functions, allows to set the specific priorities for promoting active travel across different contexts. The intricate interplay of different urban environments could shape the decision to engage in active transport. This study posits that optimal strategies will be context-dependent, requiring a nuanced understanding of how these factors interact within specific urban environments rather than being universally applicable (e.g. [33–35]),. By examining these contrasting urban settings in two cities, Greater London and Brisbane, the research seeks to identify context-specific relationships and provide insights into how urban planners can tailor interventions to prioritise effectively factors promoting active transport, considering the interplay of noise, urban form, and function.

In this paper, firstly known evidence on the relationships between active travel choices and contextual variables, such as noise pollution and the form and functions of the built environment are presented. Secondly, the methodology developed to investigate and compare non-linear associations between modal choices and contextual variables in the two cities is introduced. Finally, results are shown and discussed to then conclude by highlighting the core contributions of this work and avenues for future research.

## Literature review

Existing literature that explores what aspects of the urban context may encourage or discourage active travel has been reviewed, with a specific focus on noise pollution and the features of the built environment.

### Active travel and noise pollution

Traffic noise is common in urbanised environments and is harmful to human beings. Whilst denser neighbourhoods promote active travel, urbanisation pressure intensifies transportation needs. This contributes to adverse environment impacts where noise pollution is one of them [36].

Direct evidence suggesting noise to be a determinant of whether or not to travel actively is limited. Participants in one study identified noise to influence their route preferences but only a few indicated noise was important to them [37]. A more recent study showed a similar result but participants rated noise level to be the third most important attribute to affect comfort, which encourages or discourages active travel [38]. Pedestrians perceive traffic noise, as a result of inadequate segregation from heavy traffic to be a nuisance that reduces comfort and the motivation to walk [39]. Participants in another study perceived traffic noise to be intrusive and one of them found noise to be disrupting, which limited conversations along the journey [40]. Traffic noise decreases the willingness to walk because it makes the walking environment less attractive [41] and less pleasurable [42]. Similarly, more than half of the participants reported traffic noise to be an unpleasant factor, although the effect of noise was insufficient to determine the choice of walking routes [43]. Nonetheless, noise seems to compromise walking experience and therefore, a potential determinant.

Exposure to traffic noise could impact the quality of active travel and the travelling behaviour but evidence is indirect to relate to the choice of active travel. Pedestrians, cyclists and public transport users are frequently exposed to higher noise level compared with car users [44,45]. In an experiment, participants who were exposed to traffic noise walked relatively faster compared with those who were in the control group or listening to bird songs [46]. Assessing the relationship between noise exposure and the choice of active transportation mode is challenging because pedestrians and cyclists are typically unaware of their noise exposure level [18,47,48] as people will be habituated with decreased annoyances when exposed to noise over a longer period of time [49]. Nonetheless, a systematic review shows that cyclists are exposed to more noise than other transportation modes and in some studies of the review, cyclists perceive noise pollution as a deterrent and develop avoidance strategies [19]. Noise is perceived as a form of disturbance of comfort. Individuals with high noise sensitivity are likely to amplify stress and discomfort in noisy environments [50], making them less likely to choose transportation options that increase their noise exposure. Whilst pedestrians are more flexible with their routes to destination, cyclists could be more vulnerable to traffic noise. A quieter en-route experience available by talking could be a motivation for walking instead of cycling or commuting even by cars. Therefore, understanding the role of noise in the mode of active transportation warrants attention in research.

### Active travel and the built environment

A number of built environment attributes are identified in research to impact active travel. Higher density and diversity in urbanised settings were recurrently found to increase the likelihood of active travel [51,52]. These settings, with higher accessibility to access a number of destinations for work, shopping, leisure, and transportation in shorter distances, are associated with a higher probability of walking and cycling [53].

For results specific to the relationship between the built environment and walking, land-use diversity was the strongest predictor for walking when examining the effects of density, diversity and design on transportation modes [54]. A meta-analysis concluded that land-use diversity, intersection density and destination accessibility were the most influential factors in encouraging walking [55].

Density also plays a part in influencing cycling patterns, but the evidence is more indirect. A higher population density is associated with a higher probability of cycling [56,57]. The observation could be due to the need for greater diversity in local destinations and reduced distances between places to support the dense population. Similarly, mixed land use tends to shorten trip distances by bringing origins and destinations closer together [56,58]. Thereby, neighbourhoods with a greater mix of land uses make commuting by bike more convenient [59]. In terms of urban design, some studies observe a higher level of street connectivity, could increase the chance of bicycle use [58–62].

## Materials and methods

This study embraces a comparative approach by analysing two different urban contexts, Greater London and Brisbane. These two cities have been selected for two reasons: on the one hand they provide contrasting urban environments, which allows us to consider and discuss whether and how the relationship between noise pollution and travel choices varies in different contexts; on the other hand, Greater London and Brisbane provide data rich cases with very similar noise mapping strategies and origin destination commuting data with information on modes of transport.

Greater London, the capital of the UK with ~8.8 million inhabitants and a population density of ~ 5600 people per square km, is investigated beside Brisbane, Queensland’s capital (Australia), which plays a key role in the region but has a much lower population (~1.28 million) and population density of ~ 950 people per square km. Road infrastructure in Greater London is in general more spread out compared with Brisbane (Supporting Information S1). Analysing these two cities aims at uncovering the specificities and commonalities of travel behaviours across different contexts in terms of population size and, importantly, density, under the constraint of data availability. In particular, the role that noise and other contextual variables play in influencing people’s mode of travel choices is investigated.

### Data

The study leverages a variety of data sources to capture information about the built environment, noise levels and travel behaviours. Building and street density are considered in the analysis as they are known to influence active travel [63–65]. Building areas for Greater London are sourced from the UK Buildings dataset, an Ordnance Survey product that covers buildings in Great Britain. For Brisbane, building areas are provided by the countrywide open building footprints dataset of Bing Maps. This is obtained by a two-stage process: semantic segmentation which recognises building pixels from the aerial image using Deep Neural Networks, and polygonization, which transforms building pixel blobs into polygons. Both building datasets comprise vector polygons with information on the shape area. These areas are intersected with a regular hexagonal grid for each city and summed. Regarding road information, the study solicits OS Open Roads by Ordnance Survey for Greater London and the Road Hierarchy Overlay – Road Hierarchy in Brisbane City Plan 2014 for Brisbane. Both datasets contain line vectors with a centre approximate to the centreline of roads. The sum of lengths for the roads in each grid cell is computed as a proxy for assessing street density. In terms of proxies for urban vitality, the frequency of Points of Interest (POI) by types are considered in both cities. POIs are extracted OpenStreetMap data through Geofabrik. The label of each point vector was re-categorised into one of the six categories according to use and functions: educational, recreational, medical, public services, retail and others. Details of the re-categorisation are available in SM B. The count for each re-categorised function and the total of all functions are computed for each grid.

Noise data for Greater London are provided by DEFRA via the Strategic Noise Mapping 2017. The Strategic Noise Mapping is part of the Noise Action Plan [66] aimed at assisting the management of environmental noise in the context of UK Government policy on sustainable development. The dataset indicates the calculated noise from major road, rail sources, major airport, and large urban areas with 100,000 persons and a population density equal to or greater than 500 people per km2 across England in 2017. The estimated model was cross-validated for effectiveness with Geostatistical Wizard where optimal fit. Error analysis was also conducted to ensure predicted values were close to measured ones, with highest errors in outlier measurements [67]. In this study the annual average noise levels for a 16-hour interval, from 0700 to 2300 is employed. These estimates are obtained by DEFRA converting hourly noise levels derived from the Calculation of Road Traffic Noise (CoRTN) to the 16-hour period required by the EU directive on noise measures and implemented in the UK legislation for the Noise Strategic Mapping. Noise levels for Greater London are estimated into six ordinal classes (>75.0dB, 70.0-74.9dB, 65.0-69.9dB, 60.0-64.9dB, 55.0-59.9dB, < 54.9dB).

Noise data for Brisbane are provided by Brisbane City Council via the Transport Noise Corridor Overlay in Brisbane City Plan 2014. The noise level is calculated based on road traffic using the CoRTN procedure, obtaining estimates of the annual average noise level for 18 hour-time period [68]. The dataset was validated by performing actual noise measurements and compared with the calculated noise, based on standards set out for Brisbane City Council’s Noise Impact Assessment Planning Scheme Policy and Queensland Development Code MP4.4 for buildings in transport noise corridors [69]. Five ordinal classes (<58dB, 58dB – 63dB, 63 dB - 68 dB, 68 dB - 73 dB, > 73dB) are obtained.

Commuting flows by mode of travel are captured by the 2011 UK Census and the 2017 Australian Census. These are origin-destination data, where the origins are home locations, and the destinations are workplacesThe number of commuters is disaggregated by mode of travel. This study focuses on trips by car, bicycle, and walk. All commuting data preserve privacy as they are aggregated at area level suppressing records with less than 10 observations.

### Data preparation

The analysis is carried out on a regular hexagonal grid of ~25 hectares, covering both cities. All data describing the built environment forms, such as street and building density, functions, through POI count, and noise levels, based on the estimate modelled for each city, are interpolated to the regular grid. Building area and street length are computed for each hexagonal cell as a proxy for building and street density. POIs of each type are counted and the noise category covering the majority of the cell is selected as the most representative of noise level in that area.

**Fig 1:**
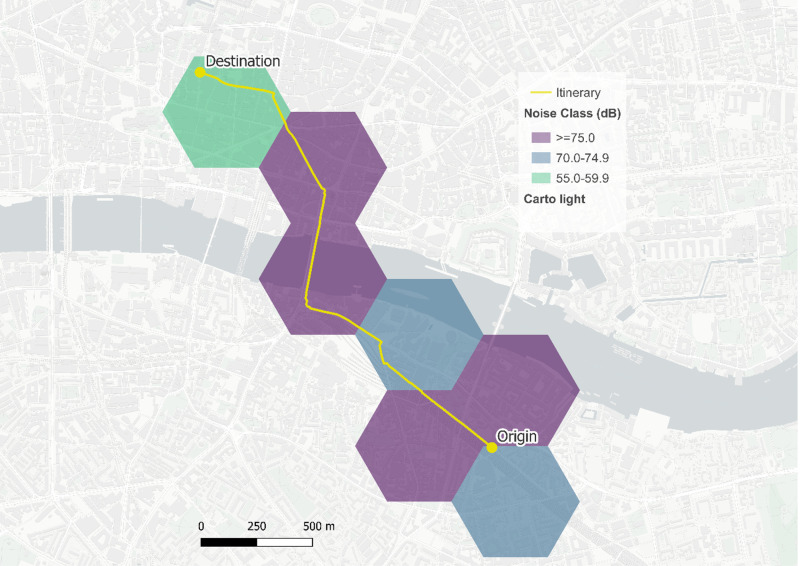
Exemplary itinerary intersecting the hexagonal grid in Greater London (anonymised) © OpenStreetMap contributors.

The street network itineraries of each commuting flow are computed through a multi-modal routing system – R5R [70] – by mode. The itineraries are then intersected with the hexagonal grids of the two cities to capture built environment forms and functions as well as the noise levels characterising each trip. Fig 1 shows an exemplary itinerary intersected with the hexagonal grid in Greater London.

Such contextual information can then be summarised, so that the resulting processed data have the following attributes: origin, destination, number of commuters travelling between the origin and the destination, mode of travel, total distance of the trip, average building area and street length of the cells intersected by the itinerary, average number of POI that the commuters have crossed in the trip by category of POI, proportion of the distance travelled in the itinerary where commuters have been exposed to each noise level. Finally, these trips are duplicated based on the number of commuters, obtaining a dataset where each row corresponds to an individual. To capture whether and to what extent these variables have an influence on modal choices a random forest model for each city is tuned, trained and tested. The choice of employing a random forest model to predict travel mode and investigate its relationship with the contextual variables listed above, is due to a number of recent studies demonstrating its high prediction accuracy and effectiveness in analysing travel behaviours [24,71,72]. More generally, random forest models can learn from mixed data types and are particularly effective for multi-classification problems [24]*,* and uncover non-linear associations between travel mode and other contextual variables [73,74].

### Random forest tuning, training and testing

**Fig 2:**
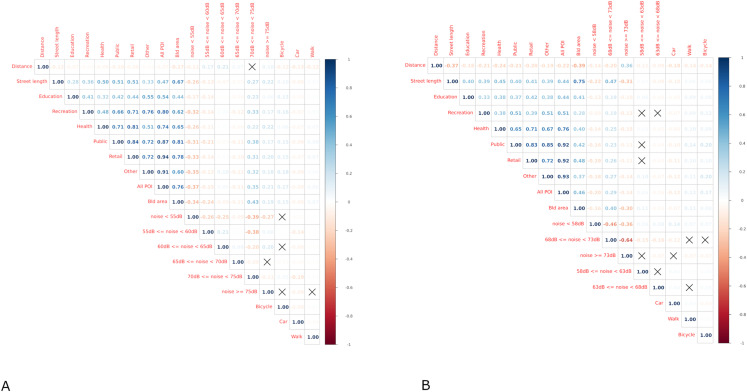
Correlation plots of trips variables in Greater London (A) and Brisbane (B).

Random forest (RF) is a supervised machine learning algorithm employed in this study to predict the mode of travel – car, bicycle, walk – from ~300k trips in Greater London and ~300k trips in Brisbane. RF combines the output of an uncorrelated forest of decision trees through bagging and random feature selection [24]. Bagging, or bootstrap aggregation, is an ensemble method where a set of random samples are selected with replacement in the training set and trained independently, to then identify the predictions that obtained the most votes – in this case the most frequent mode of travel predicted across all random samples – cross-validating the models with an out-of-bag (OOB) sample. RF implements an extension of the bagging procedure insofar as it does not only grow multiple decision trees, but also randomly selects different features for each sample in the decision tree, ensuring that these are not correlated [75]. It is important to note that, as feature importance can be affected by highly correlated data, a variable selection, removing contextual variables that have a correlation coefficient higher than 0.8, is carried out. Fig 2 shows the correlation plots of the input data for the two cities and a high correlation among types of POI can be noted.

Consequently, only retail, education, recreation, and health POI have been retained. Furthermore, since training the RF algorithm with unbalanced data is a well-acknowledged problem [76] and the majority of trips both in Brisbane and Greater London are made by car, the data used for tuning and training are first rebalanced by under-sampling. In particular, the near-miss algorithm is used on a scaled training set to down-sample all majority classes to have the same number of observations of the minority class by removing the data points in the majority classes that are more similar to those of the minority class [76].

While implementing RF, some hyperparameters need to be tuned to then train the best model [75]. A grid search on the number of decision trees and the maximum number of features selected when constructing a decision tree were performed, while the remaining hyperparameters are set to their default values. The hyperparameters tuning aims at obtaining improved model performance and generalization. S2 Fig A and B in Supporting Information show the results of the hyper-parameter tuning. Since there was not any significant accuracy improvement over 500 decision trees for both Greater London and Brisbane, the models were tested with 500 trees to find the best maximum number of features. In the Greater London model 3 resulted to be the best number of features resulting in 92% accuracy, while in the Brisbane model it was 4 with 84% accuracy. Based on these results the models are trained on 70% of the data respectively for Greater London with 500 decision trees and 3 maximum number of features for randomisation, and for Brisbane with 500 decision trees and 4 maximum number of features for randomisation. These models are then tested on 30% of the original datasets of each city that have not been employed for training. S2 Fig C in Supporting Information shows the confusion matrices for the tested models in Greater London and Brisbane. Code implementing the data exploration and modelling is available at: https://github.com/aelissa/noise_pollution.

### Variable importance and partial dependence

To interpret the results of the models two RF outputs are investigated. First, the relative variable importance, measured through the Gini importance index. This index is a measure of the mean decreased impurity gained by splits of a given variable, improving the accuracy of the model. It ranges from 0 to 1, where the higher the mean decreased impurity, the more relevant a variable is in predicting the mode of travel. Second, partial dependence plots (PDPs) are explored. PDPs show how the predicted logits (or log of the fraction of votes) for each travel mode change as the other contextual variables vary, defined according to the following partial dependence function:


$$\begin{array}{c} f(x)=logp_{k}(x)-\sum {\mathrm{\backslash nolimits}}_{j=1}^{K}logp_{j}(x) \end{array}$$ (1)

where *K* the number of classes, *k* is the predicted class, and $p_{j}$ is the proportion of votes for class *j* [77]. The plots visualise Eq. 1 for all contextual variables and modes of travel. Negative values in the y-axis mean that the log-odds of the predicted class being voted is lower than the log-odds of the other two classes. Positive values in the y-axis mean that the log-odds of the predicted class being voted is lower than the log-odds of the other two classes. Each plot represents the association between the predicted class and each predictor, controlling for the other contextual variables by holding them at their means [77].

## Results

**Fig 3:**
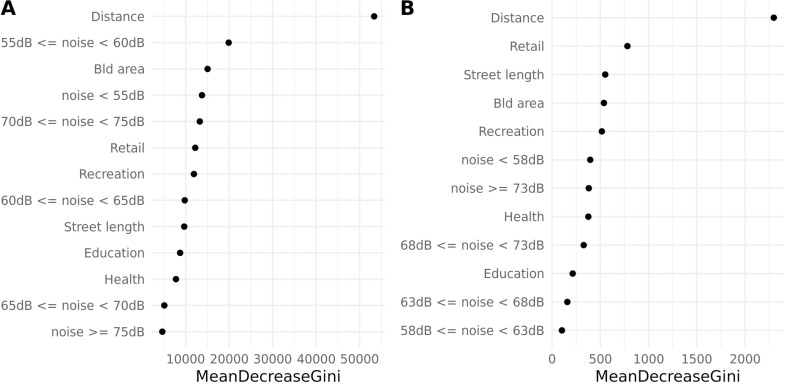
Variable importance plots for Greater London (A) and Brisbane (B). Distance is the most important variable in both cities and noise is more relevant to the mode of travel prediction in Greater London than in Brisbane.

**Fig 4:**
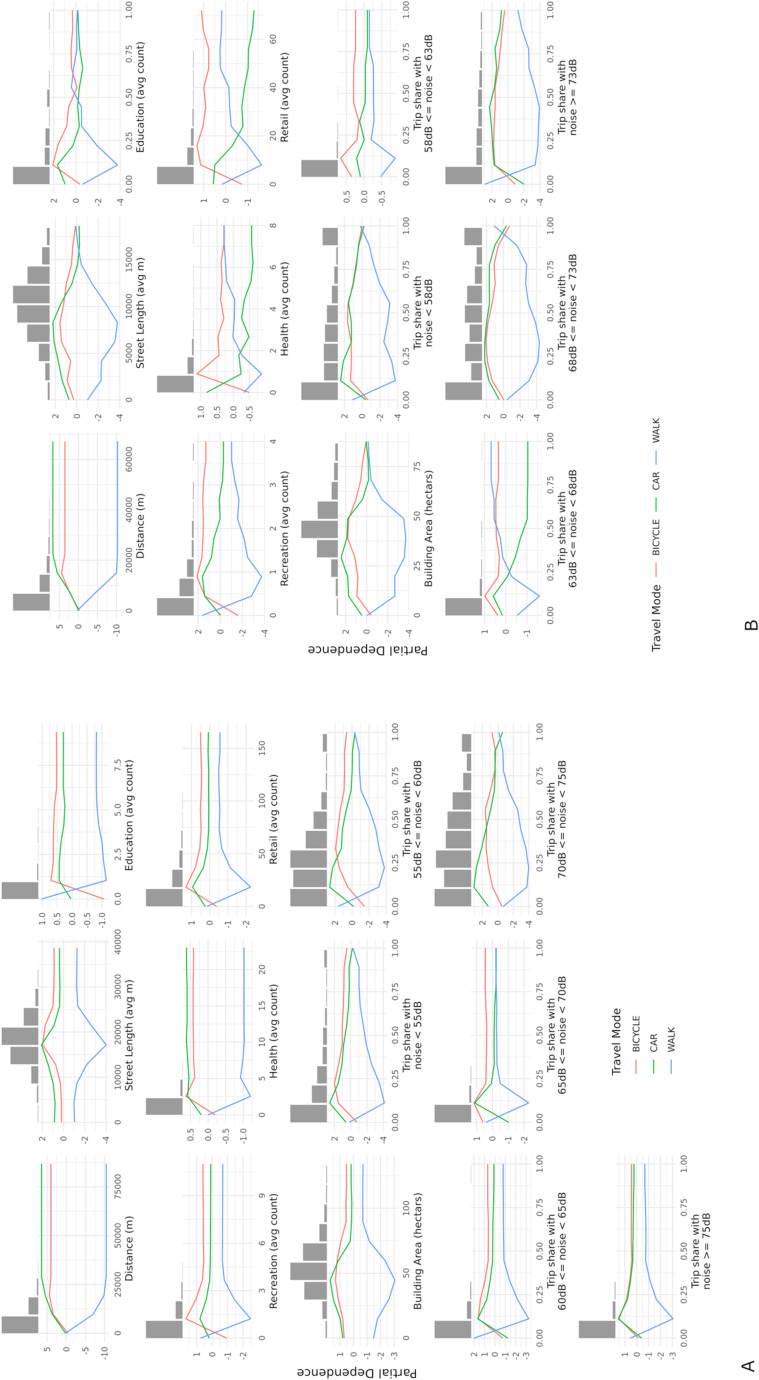
Partial plots for Greater London (A) and Brisbane (B). The log odds of predicting a trip to be made by different mode of transportations (car, bike or walking) broke down by each contextual variable (distance, street lengths, education, recreation, health, retail, building area) and trip share travelled across each noise class.

The relative importance measures of each explanatory variable are shown in Fig 3, while Fig 4 shows the partial plots for Greater London (A) and Brisbane (B).

Distance is expectedly the most important variable both in Greater London and Brisbane (Fig 3) with a clear trend that associates longer distance trips mostly with driving than cycling, while walking is predicted only for short distance trips (Fig 4). We note that noise levels are more relevant to the mode of travel prediction in Greater London than in Brisbane (Fig 3), especially when trips cross low levels of noise – either between 55db-60db and below 55db. In both cases, walking is more likely to be the predicted class when commuters are exposed to that noise level for less than 10% of the trip and for more than 90%, while driving and cycling are the more likely predicted classes with a level of exposure ranging 10% to 50% (Fig 4). Crossing higher levels of noise does not seem to be as important as crossing quieter roads, with the exception of the 70db-75db class that in the Greater London model is among the 5 most important variables despite a more significant reduction in the mean decreased impurity (Fig 3). For this class, patterns are similar to the exposure to low levels of noise for all travel modes, although the log-odds of predicting a trip to be made by car become higher than cycling for exposure over 50% as opposed to 25% exposure for lower noise classes (Fig 4). In the Brisbane model, variables related to the characteristics of the built environment play a more relevant role, while noise levels do not seem to significantly drive the model’s predictions (Fig 3). Crossing retail areas across the trip is the second most significant predictor of detecting the mode of transport (Fig 3). In particular, when commuters cross more than 10 retail points of interest on average, the likelihood of using a bike to travel becomes the highest, followed by walking when the trips cross on average 16 or more retail points of interest on; travelling by car is more likely to be predicted only with very low average counts of retail points of interest (less than 8), and such likelihood steeply decreases when the average number of retail point of interest crossed increase.

The street lengths and building areas which are proxies of street and building density respectively, hold a high and similar importance in the Brisbane model. Fig 4 shows that the associations between these two variables and the predicted mode of travel have a very similar trend: at a low level of density travelling by car is the most likely predicted mode, while when density increases first cycling and then walking become more likely.

Building density is also the third most important variable in the Greater London model (Fig 3). In Greater London, the trends characterising the association between the mode of travel log odds and the building density are similar to those seen for Brisbane, with a higher likelihood of the model predicting driving for low levels of density, followed by cycling becoming the more likely mode of travel for a higher level of density; while the likelihood of the model predicting walking increases with higher building density, differently from Brisbane, it remains below zero (Fig 4). Furthermore, Fig 4 highlights that in both cities the associations between predictors and predicted classes are always non-linear.

## Discussion

Overall, both the Greater London and Brisbane models have distance as the main determinant of modal choices. This was highly expected as distance is known to be the primary cost of moving from one place to another, and that, especially in the case of walking, the objective is to minimise it [78]. However, it is interesting to note that the likelihood of cycling follows a very similar pattern to that of driving in relation to distance. This highlights the key role of cycling in the context of sustainable mobility transitions, which demonstrates a clear potential in replacing driving for long trips. Furthermore, the likelihood of cycling long distance is apparent both in Greater London and Brisbane despite the two cities are characterised by a different climate, which could have made such pattern more likely for the latter, having higher temperatures throughout the year. This could be explained by cultural differences. In fact, London has a longer history of promoting active travel and a stronger cycling culture compared to Brisbane [79]. This established culture may influence individual perceptions and acceptance of noise exposure during cycling, as cyclists may be more accustomed to urban conditions. Brisbane, on the other hand, may exhibit a stronger car-dependent culture due to its sprawling urban form [80], leading to a greater emphasis on built environment amenities that encourage active travel as a deliberate shift away from car use. Furthermore, while Brisbane’s subtropical climate may make cycling more appealing during cooler months, the more extreme hot and humid summers could make it less attractive, increasing the reliance on air-conditioned private vehicles [81].

Noise pollution plays a more significant role in Greater London which could be due to the slight differences in which noise is estimated in the two cities. In both cases noise is predicted from traffic flows based on the Calculation of Road Traffic Noise (CoRTN) approach; however, Greater London measures have to adapt the original 18-hour averages into 16-hour averages to comply with the European directive and resulted in 6 categories of noise rather than 5. A shorter hour slot – 7AM-11PM instead of 6AM-12AM – and an additional noise category return a more detailed representation of people exposure to noise during day-to-day mobility, which could have driven the importance of this variable up in the transport mode classification of the trips. However, despite holding different importance in the Greater London and Brisbane models, noise level exhibit non-linear associations with modal choices in both also following similar trends. Walking across all categories of noise displays higher likelihood for both the low and high ends of exposure during the trip (an U shape); this is possibly due to the shorter distance characterising trips that are walked, making it more likely to cross only one category of noise, hence the proportion of exposure more proximal to 0 or 100% (see Fig 4). While results highlight that noise plays a role in modal choices, especially in Greater London, the nature of this relation takes multiple forms especially in relation to active travel. On the one hand, we note that the likelihood of cycling is systematically higher for higher level of exposure, while the relationship between walking and noise has a less linear trend likely due to the shortness of the trips as mentioned before and to the possible mediating factor of building density, which tends to drive the likelihood of walking up and at the same time correlates negatively with the lowest noise categories (−0.34 and −0.24 with exposure to <55db and 55db – 60db in Greater London, −0.16 with exposure to <58db in Brisbane) and positively with the higher noise level categories (+0.43 with exposure to 70db-75db in Greater London, + 0.4 with exposure to 68db-73db in Brisbane) (see Fig 2). Although, it is important to mention that the correlation between building density and noise takes a different sign with the highest category of noise in Brisbane (−0.3 with exposure to >73db) as that noise level mostly corresponds to the motorway and some major roads that bypass the city; on the contrary, in Greater London several major roads cross the centre of the city region.

Retail is the most important variable in the Brisbane model and active travel is strongly prevalent when trips cross higher numbers of amenities. Compared to Greater London the city is smaller with a more concentrated number of amenities in the city centre which makes it more attractive for active travel modes. Furthermore, Brisbane City council limits parking to 2 hours in the business district, the area with higher density of retail, which is likely reducing the level of car traffic and encouraging more active travel. Finally, building density confirms to be an important factor in modal choices as found in related studies [82]. Both in the Greater London and Brisbane models it is found that higher level of building density corresponds to an increase in the likelihood of walking. The relation between cycling and building density however displays a trend more similar to that of driving, which has the highest log-odds corresponding to medium level of building density to gradually, although less steeply, decrease for higher density.

## Limitations and future works

This analysis is to some respects limited as a consequence of a limited data availability.

Firstly, socio-demographic variables have been demonstrated to influence people transport mode preferences [83,84]. However, in this work investigating people’s socio-demographic characteristics has not been possible since such information could not be disaggregated at the individual level for both cities. While this would have been an interesting addition to the study, we note that the models accuracy is satisfactory enough to consider results as valuable and reliable. An avenue for future research could be that of implementing methods that estimate the socio-demographic characteristics of trip-makers based on the resident population features where the trips originate, assuming that individuals reside where their trip starts. While this will allow to investigate modal choices and socio-demographic variables, such relations will still have a high level of uncertainty.

Secondly, the study is unable to capture temporal variations as both noise level data and trip data do not have temporal information. While this is certainly relevant to understand fine-grained relationship, we believe that we are still able to capture high level patterns for two reasons: we can assume that a street which is consistently more noisy than another over a xx long period, will likely tend to be more noisy across a day; as the mobility patterns we employ are commuting trips and high peaks of traffic, and consequently noise, happen at commuting time, we should be able to capture relationships when noise levels impact on daily averages more.

Finally, new evidence can emerge from the analysis of new forms of mobility data when the mode of transport is detected [85]. Analysing spatial traces rather than itineraries will allow to drop the assumption that people prefer reaching a destination through the shortest path. Such data coupled with more detailed and rich information on the quality of existing active travel infrastructures will result in a more realistic representation of travel preferences. Furthermore, new measures of noise levels are in the process of being published in the UK which will allow updating the work.

## Conclusion

Active travel is fundamental for a healthy lifestyle [1] and in the context of sustainable mobility transitions [86].

In this study, we provide new evidence on the relationships between modal choices and noise pollution leveraging on a new spatially informed quantitative approach. Compared to existing works, rather than analysing aggregated origin-destination flows as they would happen on straight lines, we generated routed itineraries and studied exposure to noise pollution and contextual information throughout the journeys.

We show that walking and cycling are both influenced by distance, urban forms and functions, and noise pollution but in a different way. Cycling is less sensitive to farther distances and seems to have a systematically higher exposure to noise pollution; furthermore, although cycling is identified by the models as more likely than driving in highly dense and well served urban areas, it does not follow the upward trend displayed in walking behaviours, whose likelihood significantly increases when building density and amenities, especially retail, increase. It is therefore important to recognise these differences in the definition of policies aimed at encouraging active travel as they require varying interventions. For example, given the importance of density and amenities for walking, interventions such as pedestrianisations are likely more successful in well-served areas; while to encourage cycling the priority should likely go to the provision of proper infrastructures that protect cyclists from overexposure to noise pollution. Furthermore, urban policies as the 20- or 15- minute city [87–89] have a higher relevance in the context of promoting walking behaviours, while cycling can be a sustainable mean of travel in a way that is much less dependent from distance or the time it takes to reach services but more on the quality of infrastructures.

Another point that is worth noticing is the correlation between the exposure to higher levels of noise pollution and the crossings of highly dense areas; the only exception is the highest noise level in Brisbane which mostly captures noise from motorways which bypass the city rather than crossing high density areas. This highlights the need for more work and interventions for pollution free dense urban environments, which are not only affected by noise pollution but also air pollution as other studies have demonstrated [90]. Therefore, policies such as the Low Traffic Neighbourhoods applied in highly dense and well-served contexts can be an effective mean to tackle this issue, provided that they also need to implement adjustments to avoid a disproportionate impact in job access on more disadvantaged population groups [91].

From a policy perspective, the promotion of active transport, particularly cycling, requires more than merely expanding and improving cycling infrastructure. It necessitates the incorporation of design features that address the heightened noise levels associated with increased cycling activity, especially in areas where noise pollution is already high. Notably, the likelihood of cycling is systematically higher with greater exposure to noise, indicating that cyclists are more likely to choose routes in noisier environments. Therefore, interventions such as physical noise barriers or green infrastructure designed to mitigate noise exposure for cyclists could be effective measures to accompany policies for widening cycling networks.

Urban development priorities are not universal across cities but are instead dependent on city-specific factors such as density. The contrast between Greater London and Brisbane illustrates this point. Greater London, with a higher population density experiences a more significant impact of noise on transportation mode choices compared to Brisbane, which has a much lower density. In denser urban environments like London, noise becomes a more pressing issue due to the concentration of people and activities, making it a higher priority for urban planners and policymakers. Conversely, in less dense cities like Brisbane, other factors may take precedence in shaping transportation choices and urban development strategies. This density-dependent approach is supported by research on urban sustainability. However, these benefits must be balanced against potential drawbacks such as increased noise pollution and congestion. Therefore, urban development strategies should be tailored to the specific density characteristics and challenges of each city, rather than applying a one-size-fits-all approach as suggested in recent research [92].

## Supporting information

S1 Fig Mapping of Road Networks. (PDF)

S2 Fig Model Tuning and Performance. (PDF)

S3 Table Grouping of OSM POI. (PDF)
