# Supplementary material for: Does noise pollution influence modal choices? A random forest application
Source: PLoS One. 2025 Jun 23;20(6):e0325249. doi: 10.1371/journal.pone.0325249 (PMC12184936; doi:10.1371/journal.pone.0325249)
Supplement: S1 Fig — (PDF) [file pone.0325249.s001.pdf]

S1 Fig. – Mapping of Road Networks in Greater London (A) and Brisbane (B)

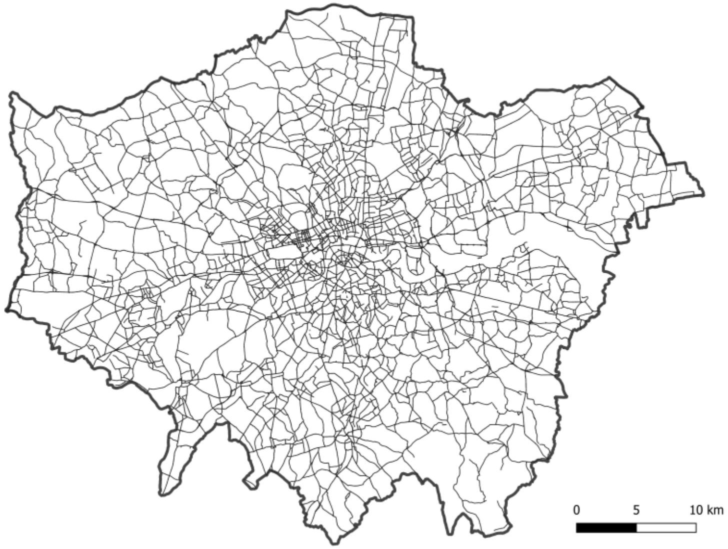

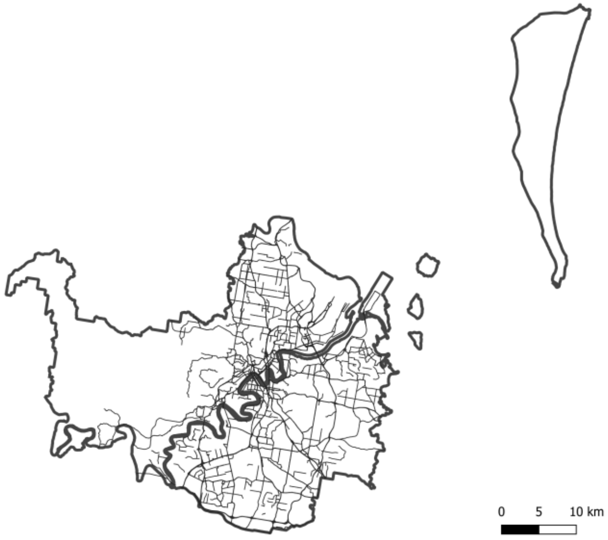

S1A Road Network in Greater London

OS Open Road Data licensed under the Open Government Licence v3.0

S1B Road Network in Brisbane

Open Data Brisbane City Council - Road hierarch11y licenced under CC BY 4.0
