# Supplementary material for: Does noise pollution influence modal choices? A random forest application
Source: PLoS One. 2025 Jun 23;20(6):e0325249. doi: 10.1371/journal.pone.0325249 (PMC12184936; doi:10.1371/journal.pone.0325249)
Supplement: S2 Fig — (PDF) [file pone.0325249.s002.pdf]

S2 Fig – Model Tuning and Performance

Tuning RF on Greater London (S2A) and Brisbane (S2B) data, to find the best number of trees (A) and max number of variables (mtry) with 500 trees based on prediction accuracy on the test data (B and C).

S2A

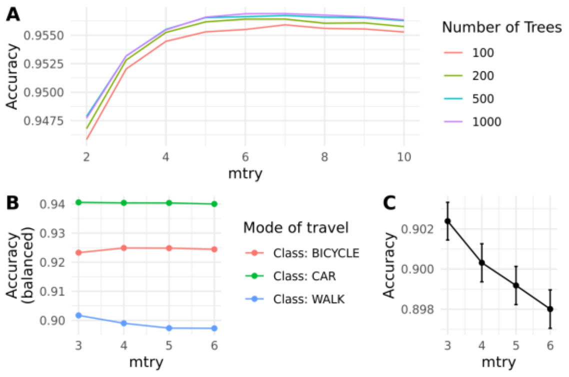

S2B

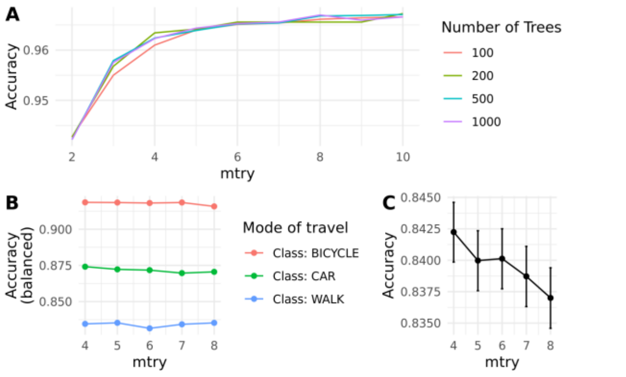

S2C – Confusion matrix and table with detailed best model performance metrics by class tested on 30% of Greater London (A) and Brisbane (B) data.

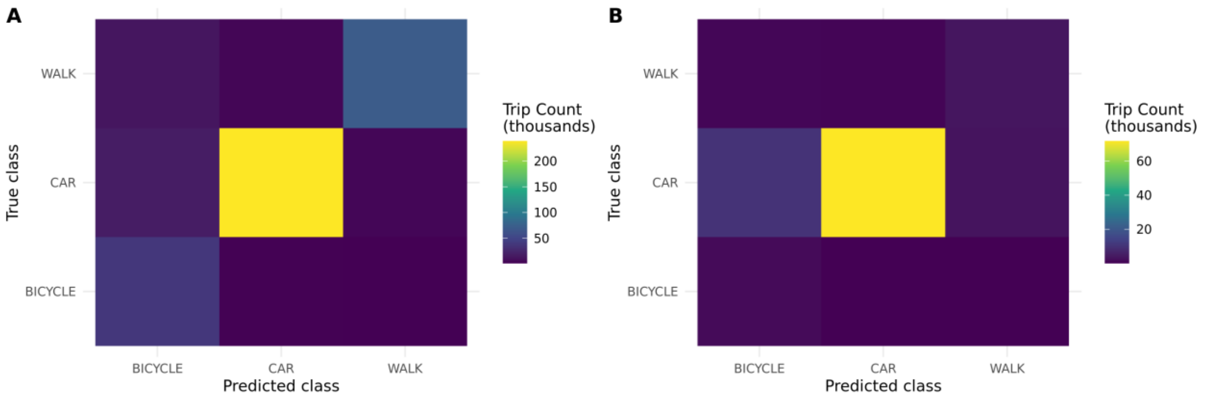

Metric (Greater London)	Bicycle	Car	Walk
Sensitivity	0.927989766	0.921833331	0.820239
Specificity	0.917967289	0.958357391	0.983173
Positive Predicted Value	0.571402603	0.978433091	0.933111
Negative Predicted Value	0.990839757	0.856783244	0.950277
Precision	0.571402603	0.978433091	0.933111
Recall	0.927989766	0.921833331	0.820239
F1	0.707294206	0.949290295	0.873042
Prevalence	0.105427109	0.672067665	0.222505
Detection Rate	0.097835278	0.619534375	0.182507
Detection Prevalence	0.171219517	0.633190333	0.19559
Balanced Accuracy	0.922978527	0.940095361	0.901706

Metric (Brisbane)	Bicycle	Car	Walk
Sensitivity	0.956123	0.848697	0.696951
Specificity	0.880484	0.896595	0.965375
Positive Predicted Value	0.120303	0.990981	0.528257
Negative Predicted Value	0.999149	0.306834	0.982836
Precision	0.120303	0.990981	0.528257
Recall	0.956123	0.848697	0.696951
F1	0.213716	0.914337	0.600991
Prevalence	0.016807	0.930493	0.0527
Detection Rate	0.01607	0.789707	0.036729
Detection Prevalence	0.133577	0.796894	0.069529
Balanced Accuracy	0.918304	0.872646	0.831163
