# Supplementary material for: Does noise pollution influence modal choices? A random forest application
Source: PLoS One. 2025 Jun 23;20(6):e0325249. doi: 10.1371/journal.pone.0325249 (PMC12184936; doi:10.1371/journal.pone.0325249)
Supplement: S3 Table — (PDF) [file pone.0325249.s003.pdf]

S3 Table – Grouping of OSM POI. Categories used in the study are in bold followed by the OSM POI names grouped under each category.

**Code**	**Education**	**Code**	**Retail and Services**
2084	college	2502	bakery
2012	community_centre	2305	bar
2083	kindergarten	2529	beauty_shop
2082	school	2518	beverages
2081	university	2542	bicycle_shop
		2515	bookshop
**Code**	**Medical/Health Care/ Clinics**	2516	butcher
2111	clinic	2303	cafe
2514	chemist	2541	car_dealership
2121	dentist	2563	car_rental
2120	doctors	2564	car_wash
2101	pharmacy	2512	clothes
2110	hospital	2546	computer_shop
2519	optician	2511	convenience
2129	veterinary	2505	department_store
2013	nursing_home	2543	doityourself
		2302	fast_food
**Code**	**Cultural and Recreation**	2513	florist
2014	arts_centre	2306	food_court
2203	cinema	2544	furniture_shop
2257	ice_rink	2547	garden_centre
2007	library	2521	gift_shop
2722	museum	2528	greengrocer
2251	sports_centre	2561	hairdresser
2253	swimming_pool	2405	hostel
2201	theatre	2401	hotel
2008	town_hall	2520	jeweller
2743	zoo	2503	kiosk
2744	theme_park	2568	laundry
		2504	mall
**Code**	**Public Use**	2525	mobile_phone_shop
2601	bank	2402	motel
2009	courthouse	2527	newsagent
2011	embassy	2202	nightclub
2002	fire_station	2524	outdoor_shop
2001	police	2304	pub
2005	post_office	2301	restaurant
2010	prison	2517	shoe_shop
2099	public_building	2522	sports_shop
2030	recycling	2523	stationery
2510	general	2501	supermarket
		2526	toy_shop
		2567	travel_agent
		2530	video_shop
		2404	guesthouse
		2406	chalet
